# Growth of cholesteatoma by implantation of epithelial tissue along the femoral bone of rats

**DOI:** 10.1016/S1808-8694(15)31309-4

**Published:** 2015-10-20

**Authors:** Sandra Lira Bastos de Magalhaes, Olga Maria Rojas Reforme, Raquel Liriano Guzmán, Yotaka Fukuda, Flávia Barbosa

**Affiliations:** ^1^ Otorhinolaryngologist; ^2^ Resident physician, Service of Clinical Pathology, Hospital do Servidor Público Estadual (Pathologist); ^3^ Physician, Post-graduation studies under course, Program of Otorhinolaryngology and Head and Neck Surgery, Federal University of Sao Paulo - Escola Paulista de Medicina- UNIFESP - EPM/SP (Otorhinolaryngologist); ^4^ Associate Professor, Full Professor, Discipline of Otorhinolaryngology, Federal University of Sao Paulo - Escola Paulista de Medicina - UNIFESP-EPM/SP; Otorhinolaryngologist, Hospital IGESP (Associate Professor, Full Professor, Discipline of Otorhinolaryngology, Federal University of Sao Paulo - Escola Paulista de Medicina); ^5^ Resident Physician, Discipline of Otorhinolaryngology, Hospital do Servidor Público Estadual (Otorhinolaryngologist). UNIFESP - Escola Paulista de Medicina - Sao Paulo

**Keywords:** cholesteatoma, epidermoid cyst, middle ear otitis

## Abstract

Cholesteatoma is a well-known infection resembling a pearl. Its histological aspect is of an epidermal cyst formation characterized by epidermal-keratinized tissue in the middle ear and mastoid that can migrate and erode to adjacent structures.

**Aim:**

To verify epidermal cyst (cholesteatoma) growth through implantation of auricular skin of a mouse next to its femoral bone.

**Study design:**

experimental.

**Material and Method:**

Ten healthy rats between two and five months of age and of both sexes underwent implantation of auricular skin on the femoral bone during a three-month period. Paraffin-embedded sections were obtained from the sample and stained with hematoxylin and eosin for pathology investigation.

**Results:**

Macroscopic view: round soft yellowish granulation tissue. Microscopic view: keratinizing stratified squamous epithelium cystic formation. The cyst presented innermost corneal layer, resulted from keratinized skin, followed by granulated and squamous layers, and outermost basal layer. **Conclusions**: Growth of epidermal cyst (cholesteatoma) may start from a transplanted epithelial tissue next to the femoral bone of rats.

## INTRODUCTION

Cholesteatoma is a well-known infection of the middle ear and was firstly reported by Curveilhier in 1829 as a pearled tumor. In fact, cholesteatoma is a squamous cell cyst, characterized by keratinized epidermoid tissue that can migrate and erode to adjacent structures frequently found in temporal bones and in people with history of chronic middle ear otitis^1^, ^2^.

The squamous cell cyst presents an external matrix formed by keratinizing stratified squamous epithelium over a perimatrix of fibroconnective tissue (connective tissue containing collagenous, elastin and reticulin fibers, lymphocytes, histiocytes and plasmocytes, besides newly formed vessels). Histologically, the epithelium is similar to the epidermis and its four basic layers can be observed (basal, squamous, granulated and corneal). The matrix peels off keratin scales (lamellas) into its internal own area, filling and enlarging it^3^.

Cholesteatoma has lytic and migrating characteristics and it may destroy both the ossicular chain and the mastoid bone cavity, leading to intra and extracranial complications.

Traditionally, this cyst is classified as congenital and acquired (primary and secondary), depending on its genesis. Pathogenesis of acquired cholesteatoma is directly related to middle ear affections, either by dysfunctions or infections of the auditory tube, leading to perforation of the tympanic membrane, which is not observed in congenital cholesteatoma.

Recent studies demonstrated that the onset and evolution of cholesteatoma seem to be multifactorial and are related to genetic and environmental characteristics, including aspects of molecular biology, once cytokeratins are present in epithelial cells with latent features of proliferation and migration. Moreover, a cascade performance of cytokines – proteins produced by cells in response to an inflammatory process modifying their own cellular characteristics or surrounding tissues^4^. This interaction between cytokines is, at the same time, cause and effect of the aggressive feature of cholesteatoma^3^.

Given it is an epidermal tissue, the cholesteatoma has cytokeratins 1, 2, 10 (typical of keratinizing stratified epitheliums) and cytokeratins 5 and 14 (typical of keratinizing stratified or non-keratinizing epitheliums^3^.

Cholesteatoma is diagnosed by an accurate anamnesis, while history of disease is essential due to its peculiar features. At otorhinolaryngology examination, otoscopy should be performed microscopically. Attention should be given to presence of secretion in the meatus and tympanic cavity, which must be treated and fully aspirated. Aspect of the tympanic membrane should be observed, particularly the *pars flaccida*. Imaging should include simple X-ray, computed tomography (exam of choice) and magnetic resonance (not usual). Surgical treatment is generally adopted.

Experimental trials showed formation of epidermoid cyst when keratinized epithelium is implanted in bones^5^.

Injection of propyleneglycol in the tympanic bulla of rats induces formation of cholesteatoma probably due to migration of tympanic basal epithelium layer^6^.

Eventual reproduction of an epidermoid cyst in more accessible regions allows us to conduct a detailed study of disease onset and development, involving several molecular aspects. The thigh of a mouse was chosen as it is an accessible bone of manageable dimensions. Also, it does not involve vital structures and yields easy recovery of the animal after the procedure.

This study aimed at verifying the growth of an epidermoid cyst (cholesteatoma) after implantation of a skin fragment from the auricular pavilion to the femoral bone of rats.

## MATERIAL AND METHODS

Ten healthy rats aged 2 to 5 months of both sexes were selected and submitted to implantation of skin fragment from the auricular pavilion to the thigh. In the first three animals, only one paw received the implantation, and the others received skin grafts on both paws, resulting in 17 studied paws.

The animals were maintained in lab cages at the Embryology Lab of the Morphology Department of Federal University of Sao Paulo, where they were fed, hydrated and kept under appropriate temperature. They were assisted by a sector employee and overseen by researchers.

Animal manipulation started with general anesthesia with ether.

A skin fragment measuring approximately 4 × 4 mm was incised from a non-hairy area of the auricular pavilion. Incision of the thigh mid third was performed and the musculature was directed up to the femur. At this site, the periosteum was removed and part of the skin fragment was implanted over. After that, skin suture was performed using a 0-4 mononylon thread.

For comparative criterion, in the right thigh, the epithelium's side was placed in contact with the femur and, from the fourth animal on, the subcutaneous side of the left thigh was placed in contact with the bone.

Once the procedures were over, the animals were put back in their cages and maintained in the same environment for 3 months. After this period, the animal was sacrificed and the implanted tissue was removed. Each fragment was separately fixed according to Bouin's procedure, paraffin-embedded and stained with hematoxylin and eosin (H-E). The slides were prepared and sent for clinical pathology.

## RESULTS

### Macroscopic Aspect

Out of 17 paws, 16 presented epithelial tissue along with muscle, showing evidences of bone tissue migration to outermost layers.

No implanted tissue could be found on 1 paw.

Tissues' aspect was granulated, yellowish, round and soft, measuring about 3 mm in diameter.

### Microscopic Aspect

**Figure 1:**
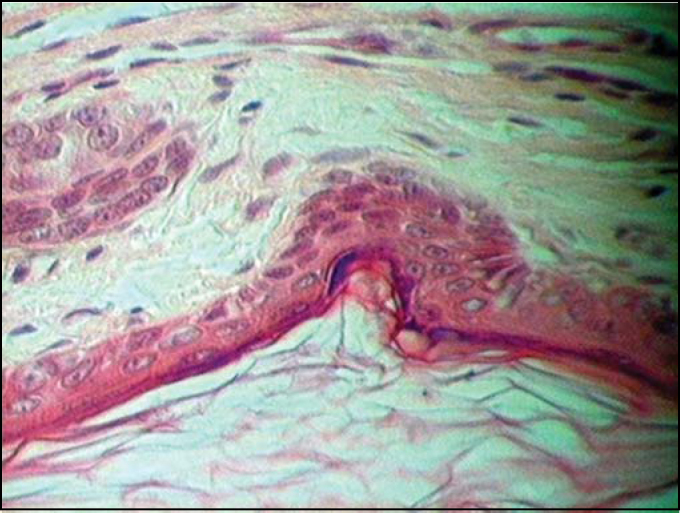
Epidermoid cyst – Innermost corneal layer (lower figure) followed by granulated, squamous and basal layers.

**Figure 2:**
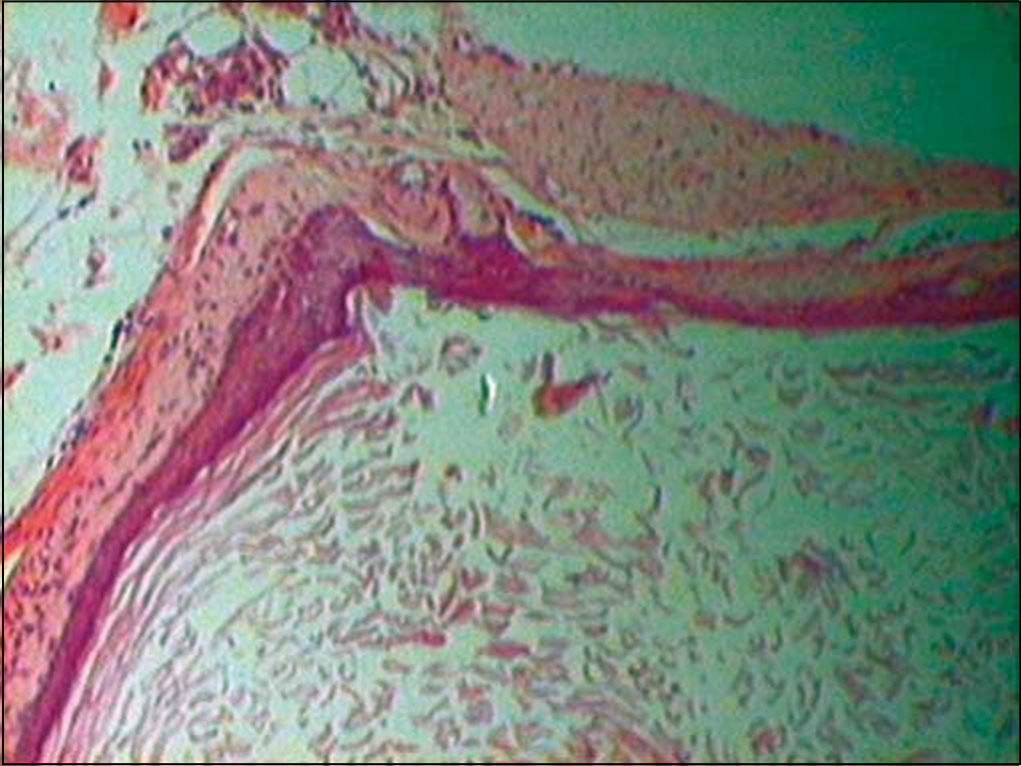
Epidermoid cyst – cystic structure coated by a stratified squamous epithelium. Innermost corneal layer (lower figure) with keratin desquamation in lamellas followed by granulated layer and, outermost squamous and basal layers.

Histological H-E-stained sections showed a cystic structure covered by squamous stratified epithelium (Figure 1-2). Corneal layer forms the cyst's innermost region with keratin desquamation in lamellas inside the cystic cavity, followed by granulating layer and externally by squamous and basal layers.

## DISCUSSION

Cholesteatoma is a destructive lesion of the temporal bone that gradually expands and leads to complications by erosion of the bone surrounding structures. Bone reabsorption may result in destruction of the ossicular chain and optical capsule followed by hearing loss, vestibule dysfunction, facial muscle paralysis and intracranial complications. Surgery is the treatment of choice. However, cholesteatoma pathogenesis is rather controversial^1^.

Studies indicate that the epidermoid tissue of the middle ear is originated in the epidermis of the external auditory canal^4^.

Epidermoid formation was again investigated in fragments of temporal bones in order to find a relation with congenital cholesteatoma. The findings confirm that the epidermoid tissue is precursor of the congenital cholesteatoma^7^.

A similar study was conducted in rats, although skin implantation of external auditory meatus was transplanted in the middle ear. The result was formation of an epithelial cyst or gradual transition in the middle ear epithelium. In addition, expressive epithelial growth was observed when over imposed infection occurred^8^.

Another investigation with the same characteristics of our research study had also verified formation of epidermoid cysts (cholesteatoma) in part of animals, presenting inflammatory reaction that was associated with formation of cholesteatoma^5^.

Otology procedures have been described as etiological factors in development of epidermoid cyst of the middle ear and the tympanic membrane. Case reports present history of cyst formation on the same side of otological surgeries, postulating that it may arise from epidermal elements and that implantation of keratinized epithelium may have occurred in the middle ear at the time of surgery^9^.

In trials, pathogenesis of cholesteatoma was also predominantly associated with bone reabsorption induced by a cyst and mediated by osteoclastic activity occurring within the middle ear. It is important to emphasize that this osteoclastic activity is a criterion to understand the cholesteatoma^10^, ^11^. For pathogenesis investigation issues, a skin fragment was implanted in rats. The result was an expressive increase of cytokines M-CSF, OPG and OPGL demonstrated by immunohistochemistry.

The study revealed basic events of osteoclastic activity with localized bone reabsorption and promoted new findings for understanding induced resorption by cholesteatoma^11^.

In Antunes' study^6^, there was increase of choleasteatoma by injection of propyleneglycol in the tympanic bulla of guinea pigs, without perforation of tympanic membrane. This fact emphasizes that growth of epidermoid cyst may be induced presumably by migration of innermost layers of the tympanic membrane epithelium into the bulla.

In the same study, the author used trans-retinoic acid in one of the ears of these animals, keeping the other side as control. On the side that received trans-retinoic acid, growth of cholesteatoma was very reduced, indicating that this drug should inhibit growth process of cholesteatoma^6^.

In our study, only 1 out of 17 paws did not show a cyst, which may be explained by possible absorption or migration to a distant region. On the other 16 paws with epidermoid cysts, they were rounded, soft and yellowish. The material was not found next to the femur and did not present bone erosion by the cyst, but was rather found superficially on the muscular layer, indicating that the organism was trying to expulse the cyst. Regarding the epithelium in contact with the bone, no difference in the behavior of skin or subcutaneous superficial layer was observed, leading to formation of a cyst without bone lesion. Skin fragment encysting to its uppermost layer inside the cyst shows that the organism tries to isolate keratin as if it was a foreign body.

In the last decade, the osteoclastic activity was broadly discussed as the main factor to induce bone destruction in chronic middle ear otitis due to cholesteatoma. Cellular inflammatory factor, such as cytokines, could play a role as a trigger for onset of osteoclastic activity. Following, local concentration of lipopolysaccharides could make cytokines locally available. This fact turns local concentration of lipopolysaccharides an important factor for bone resorption in the cholesteatoma^12^.

A study covering expression and distribution of transforming growth factor alpha (TGF) in the middle ear suggests that autocrine and paracrine stimulation of TGF in the cholesteatoma squamous epithelium may contribute for its proliferation^13^.

Since epidermoid cyst may be experimentally reproduced in accessible sites, molecular aspects in the formation and growth of cholesteatoma may be investigated.^14^

## CLOSING REMARKS

We came to the conclusion that the epidermoid cyst (cholesteatoma) may arise from an epithelial tissue transplanted onto femoral bone of rats. This study in experimental models shows that internalization of keratinized epithelium (skin) stimulates the organism's defense mechanism to isolate the foreign tissue to the center, forming a cyst to expel it.
